# App-Based Versus Standard Six-Minute Walk Test in Pulmonary Hypertension: Mixed Methods Study

**DOI:** 10.2196/22748

**Published:** 2021-06-07

**Authors:** Dario Salvi, Emma Poffley, Lionel Tarassenko, Elizabeth Orchard

**Affiliations:** 1 Institute of Biomedical Engineering Department of Engineering Science University of Oxford Oxford United Kingdom; 2 Department of Cardiology Oxford University National Health Service Foundation Trust Oxford United Kingdom

**Keywords:** cardiology, exercise test, pulmonary hypertension, mobile apps, GPS

## Abstract

**Background:**

Pulmonary arterial hypertension (PAH) is a chronic disease of the pulmonary vasculature that can lead to heart failure and premature death. Assessment of patients with PAH includes performing a 6-minute walk test (6MWT) in clinics. We developed a smartphone app to compute the walked distance (6MWD) indoors, by counting U-turns, and outdoors, by using satellite positioning.

**Objective:**

The goal of the research was to assess (1) accuracy of the indoor 6MWTs in clinical settings, (2) validity and test-retest reliability of outdoor 6MWTs in the community, (3) compliance, usability, and acceptance of the app, and (4) feasibility of pulse oximetry during 6MWTs.

**Methods:**

We tested the app on 30 PAH patients over 6 months. Patients were asked to perform 3 conventional 6MWTs in clinic while using the app in the indoor mode and one or more app-based 6MWTs in outdoor mode in the community per month.

**Results:**

Bland-Altman analysis of 70 pairs of conventional versus app-based indoor 6MWDs suggests that the app is sometimes inaccurate (14.6 m mean difference, lower and upper limit of agreement: –133.35 m to 162.55 m). The comparison of 69 pairs of conventional 6MWDs and community-based outdoor 6MWDs within 7 days shows that community tests are strongly related to those performed in clinic (correlation 0.89), but the interpretation of the distance should consider that differences above the clinically significant threshold are not uncommon. Analysis of 89 pairs of outdoor tests performed by the same patient within 7 days shows that community-based tests are repeatable (intraclass correlation 0.91, standard error of measurement 36.97 m, mean coefficient of variation 12.45%). Questionnaires and semistructured interviews indicate that the app is usable and well accepted, but motivation to use it could be affected if the data are not used for clinical decision, which may explain low compliance in 52% of our cohort. Analysis of pulse oximetry data indicates that conventional pulse oximeters are unreliable if used during a walk.

**Conclusions:**

App-based outdoor 6MWTs in community settings are valid, repeatable, and well accepted by patients. More studies would be needed to assess the benefits of using the app in clinical practice.

**Trial Registration:**

ClinicalTrials.gov NCT04633538; https://clinicaltrials.gov/ct2/show/NCT04633538

## Introduction

Pulmonary arterial hypertension (PAH) is a progressive illness that can be a severe life-limiting condition if not diagnosed early or left untreated [1]. It is a chronic disease of the pulmonary vasculature, with vascular proliferation and remodeling of the small pulmonary arteries leading to a progressive increase in pulmonary vascular resistance. This ultimately results in right heart failure and premature death [2]. The predominant symptom of PAH is dyspnea on exertion, with a decrease in exercise capacity. PAH is an uncommon condition, affecting about 6000 patients in the United Kingdom [3].

The 6-minute walk test (6MWT) is a standard method for measuring exercise capacity in patients with cardiopulmonary disease such as PAH. The 6MWT measures how far a patient can walk in 6 minutes [4]. Walking is an activity performed every day by most patients except for those most severely limited. By assessing patients’ ability to exercise, the 6MWT provides a global assessment of respiratory, cardiovascular, neuromuscular, and cognitive function. The 6MWT does not differentiate what limits the patient nor does it assess maximal exercise capacity. Instead, the 6MWT allows the patient to exercise at a daily functional level and is a useful tool for assessing severity of disease, and increasing walk distance correlates with a subjective improvement in dyspnea [5].

In PAH, the 6MWT is used to assess patients’ risk of death, with a walked distance of >440 m being associated with a low risk (<5% a year estimated risk of mortality ) and <165 m being a high risk (>10% risk of mortality) [6]. An increase in 6MWT distance of more than 42 m is considered a clinically significant improvement [7]. Furthermore, change in 6MWT distance correlates with VO_2_ max, New York Heart Association class, and mortality in PAH patients, providing an objective assessment of disease progression, prognosis, and response to treatment [8]. It is a universally accepted test as it is safe and easily performed by the patient.

The 6MWT has become the primary end point for many trials and as a surrogate for invalidated survival outcome for all placebo-controlled trials of PAH therapy [9]. It is used by regulatory bodies to determine whether a treatment should be approved. The test is usually performed in the hospital, by having the patient walk along a hospital corridor, where many factors on the day of the test can affect patient performance, including tiredness, lack of familiarity with the environment, or anxiety. Two physiologists are typically required to monitor the test to measure distance walked and oxygen saturations by pulse oximetry and record symptoms felt during the test. This justifies the need for a community-based approach that would allow a more accurate and frequent measure of the day-to-day function of PAH patients while reducing employed resources.

Community-based 6MWTs have been performed in chronic stroke patients using a GPS tracker [10] and in heart failure patients using accelerometers and step counters [11]. A smartphone-based method was used by Ata et al [12] for assessing patients with peripheral artery disease, but it relied on the embedded distance measurement of the iPhone and proved to have low accuracy. More promising results were achieved using a custom smartphone app in congestive heart failure and pulmonary hypertension participants [13] or our app, named SMWT, which demonstrated high accuracy and user acceptance in lab tests [14].

In this study, we aimed to test our app on 30 patients with pulmonary hypertension. The aim of the study was to assess if patients were able and willing to use the app, compare the 6MWT distance (6MWD) measured by the app with the reference measured in the clinic by physiologists, and explore the feasibility of pulse oximetry during app-based 6MWT.

## Methods

### Approach

**Figure 1:**
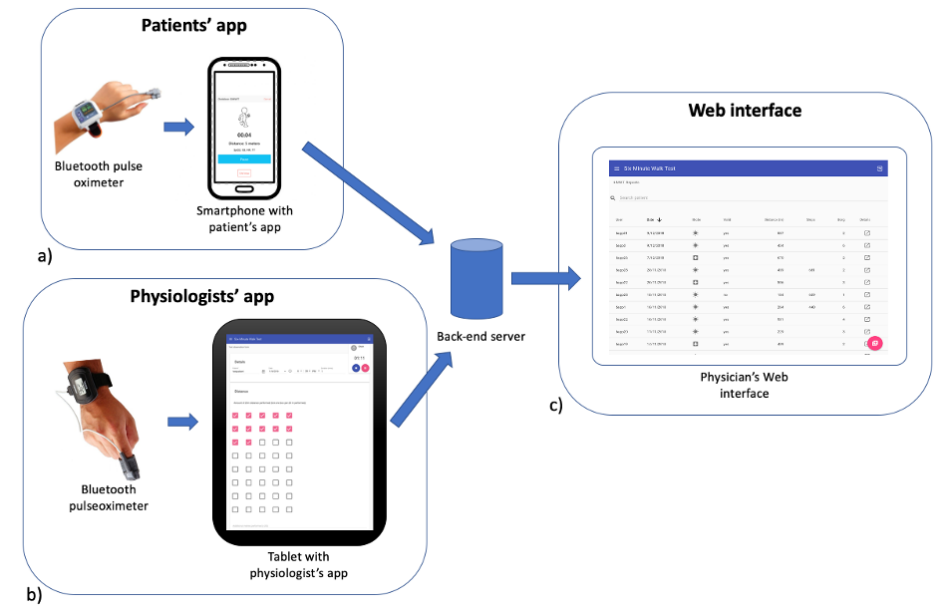
Architecture of the SMWT system: (a) patients’ app, (b) physiologists’ app, and (c) web interface for physicians.

The SMWT system is described in detail in Salvi et al [14]; nonetheless, a summary of its architecture and functionalities is provided here for reference. The system comprises a mobile phone app for patients, tablet app used by physiologists, and server (Figure 1). The patient app can work in two modalities: indoor, where inertial sensors are used to measure the number of U-turns the patient performs while walking on a straight walkway, and outdoor, where the positioning system, like the GPS, is used to track the user and compute the walked distance. The app also allows users to connect to a Bluetooth pulse oximeter, supporting both the WristOx_2_ 3150 (Nonin) and the PC-68B (Shenzhen Creative Industry Co Ltd) medical sensors.

The server collects the 6MWT data from the app and provides a web interface to review the data. Physiologists can also add conventional 6MWT information like total walked distance and symptoms on the patient’s web page. The physiologist app offers the same interface as the website, with the added possibility to collect live data from the WristOx_2_ 3150 pulse oximeter.

We defined a study protocol with the aim of demonstrating that patients are able and willing to use the app for the 6MWT. The main outcomes were percentage of participants who performed at least 1 community 6MWT and percentage of patients who provided at least 1 community 6MWT per month during the study period. Secondary outcomes were as follows:

- Comparison of the indoor app-based 6MWD against simultaneous clinic 6MWD (statistics of differences, Bland-Altman analysis)
- Comparison between community-based outdoor 6MWD and clinic 6MWD within 7 days (statistics of differences, Bland-Altman analysis)
- Assessment of test-retest reliability of the community-based 6MWT within 7 days (statistics of differences in consecutive 6MWDs, two-way random effects model, single measures, absolute agreement intraclass correlation [15], standard error of measurement computed as standard deviation of 6MWD 
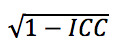
 [16], mean intrasubject coefficient of variation)
- Assessment of usability and acceptance (Mobile Application Rating Scale–user version [17] and mobile health acceptance [18] questionnaires and semistructured interviews)
- Feasibility of pulse oximetry during app-based 6MWT (Bland-Altman analysis of pulse oximetry collected by two sensors simultaneously)

### Selection Criteria

Inclusion criteria were willing and able to give informed consent for participation in the study, male or female aged 18 years and older, diagnosed with PAH but able to undertake a 6MWT off-oxygen, PAH group 1 (regular PAH) or 4 (pulmonary hypertension due to blood clots in the lungs), own a compatible smartphone (Android or iPhone) and able to use it. Exclusion criteria were use of long-term oxygen therapy; cognitive impairments; rheumatological diseases that limit the measurement of finger oxygen saturations; PAH groups 2 (pulmonary hypertension due to left heart disease), 3 (pulmonary hypertension due to lung disease), or 5 (blood and other rare disorders that lead to pulmonary hypertension); cannot use a smartphone; or pregnancy.

We focused on patients in PAH groups 1 and 4 [19] because they have treatable forms of PAH and need to perform frequent 6MWTs in order to titrate their medication regime for pulmonary hypertension and assess their response to it. Patients with cognitive impairments were excluded because they would have struggled with using the app. Patients who require oxygen to mobilize would have needed to walk and carry their oxygen supply, and this would have affected the distance that they could walk outside. Pregnancy was excluded so as to not introduce confounding factors.

Patients were recruited during regular PAH clinics. Each patient participated for about 6 months. They performed 3 conventional 6MWTs at the clinic, one on the day of recruitment, one after 3 months and one after 6 months. The clinic 6MWT was performed while having the patient’s phone running the app in indoor mode. Patients were additionally asked to perform an app-supported outdoor 6MWT in their community within 3 days of the clinic.

Between clinics, patients were invited to perform one community-based 6MWT preferably every 15 days and not less than once per month. To improve compliance, patients who did not provide a community 6MWT within 1 month were reminded either by email or phone call. At the last clinic 6MWT, participants were given usability and acceptance questionnaires [17,18] to be completed either online or on paper. A number of patients were also interviewed depending on their and the staff availability. Paper-based answers were digitized by one team member. Interviews were performed by one team member and audiorecorded with explicit signed consent. The questions were based on a technology acceptance model [20]. Audio was transcribed by one team member who also analyzed the transcriptions.

In order to guarantee quality of the collected data, both in-clinic and community-based measurements were visually assessed using the web interface by an engineer and a physiologist on a weekly basis. Tests that showed evident artefacts or that patients did not comply with recommendations about how to perform the test (ie, when the walked path showed more than 3 very narrow curves or if the variation of altitude over the entire path was more than 20 m), were flagged as invalid and not included in the analysis (examples of invalid tests are provided in Multimedia Appendix 1). The data were extracted from the database used in the system (ArangoDB) and analyzed with the R programming language (R Foundation for Statistical Computing).

The study protocol was approved by the National Health Service Health Research Authority (protocol reference number: 17/WM/0355) and registered at Oxford University Hospitals National Health Service Foundation Trust. The study was also registered at ClinicalTrials.gov [NCT04633538]. Written informed consent was obtained from all participants.

## Results

### Participants and Tests

We approached 33 patients; of these, 30 were eligible and consented to participate. Five patients withdrew from the study (3 were lost to follow-up, 1 died, and 1 considered the study not to be relevant any longer). Of the patients who started, 37% (11/30) were male and 63% (19/30) were female. Their mean age was 50 (SD 16.55; range 20-76) years. The first patient was enrolled on January 25, 2018, and the latest patient completed the study on September 7, 2019. Patients were actively enrolled in the study for a mean of 244.23 (SD 96.16; range 147-590) days.

In total, we received 455 test reports, of which 429 were considered valid according to our data quality procedures. Each patient performed 3 6MWTs in the clinic, 3 months apart from each other, using the app in indoor mode while the physiologist reported their observations (distance, oxygen saturation, Borg scale, symptoms) using the dedicated website or app. We received 81 test reports from the physiologist and 71 app-based tests in indoor mode. Between clinic visits, patients were asked to perform tests in the community using the app in outdoor mode. We received 277 such test results sent by 12 Android phones and 29 iPhones (9 patients used more than one phone during their participation in the study).

### Accuracy of the App 6MWT in Indoor Mode

**Figure 2:**
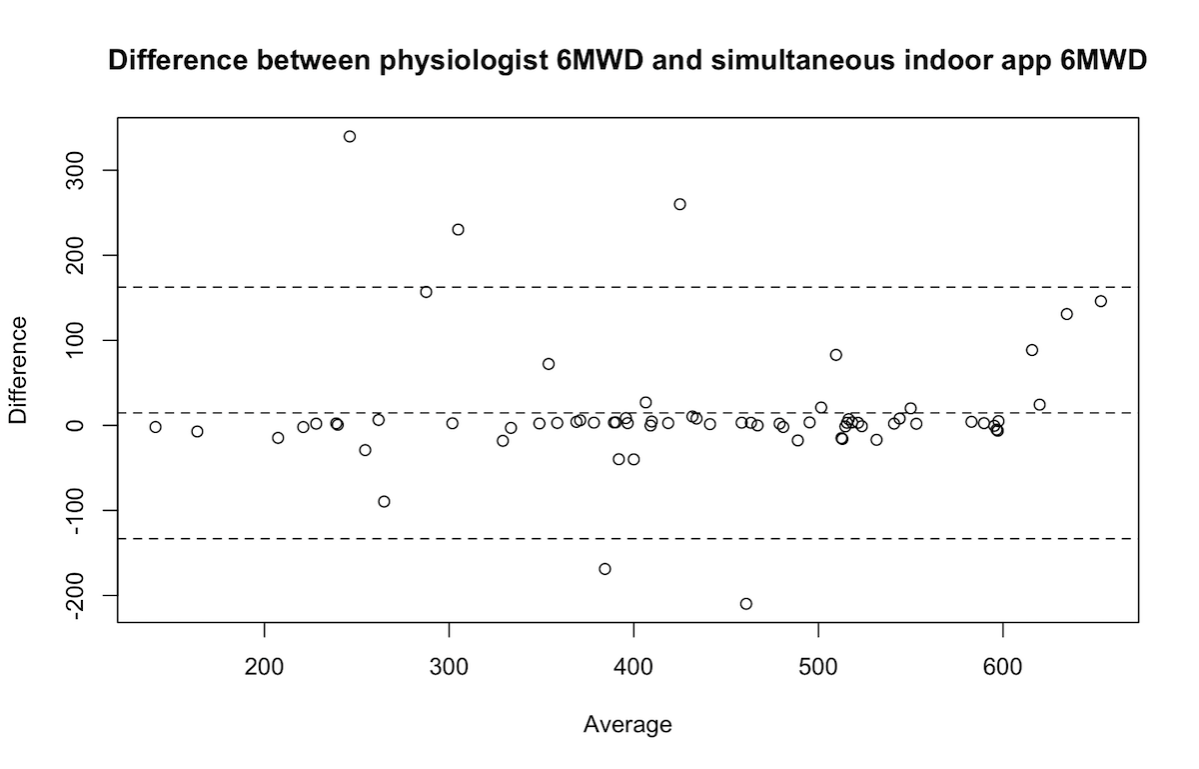
Bland-Altman plot of the difference between the 6-minute walk test distance as measured by the app in indoor mode and as observed by the physiologist during a 6-minute walk test in clinic. 6MWD: 6-minute walk test distance.

We compared 70 pairs of 6MWD, for which one was measured by a physiologist during the clinic 6MWT and the other was simultaneously computed by the app in indoor mode. The tests belong to 29 patients. To compare the two distances, we report the statistics in Table 1 and Bland-Altman plot in Figure 2.

**Figure 3:**
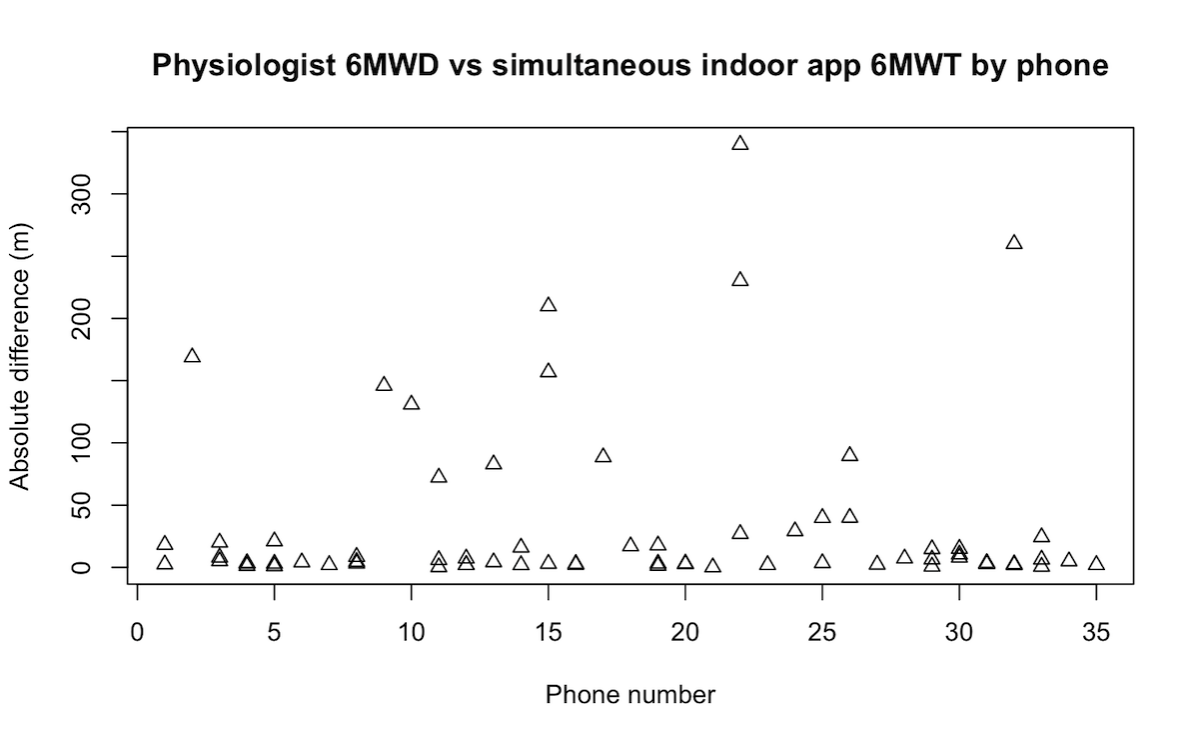
Absolute difference of the 6-minute walk test distance as measured by the physiologist and as measured by the mobile phone app in indoor mode. 6MWD: 6-minute walk test distance; 6MWT: 6-minute walk test.

To understand if the accuracy was affected by a systematic bias created by some phones (eg, because of different sensor characteristics), we plotted the absolute difference of the distance between the app-computed 6MWD and the reference value for each phone and each user in Figure 3.

**Table 1:** Differences between a 6-minute walk test distance (6MWD) measured by physiologist and simultaneous 6MWD measured by the app in indoor mode.

Characteristic	Value
Difference (m), mean (SD)	14.6 (75.48)
Difference (% of total length), mean (SD)	1.92 (18.6)
Difference (m), median (min-max)	2.65 (–209.8 to 339.64)
Difference (% of total length), median (min-max)	0.65 (–58.93 to 81.64)
Correlation (*P* value)	0.83 (<.001)
Percentage of difference below 42 m (%)	83
Lower and upper limits of agreement (m)	–133.35 to 162.55

### Validity of Community-Based Outdoor 6MWD

**Figure 4:**
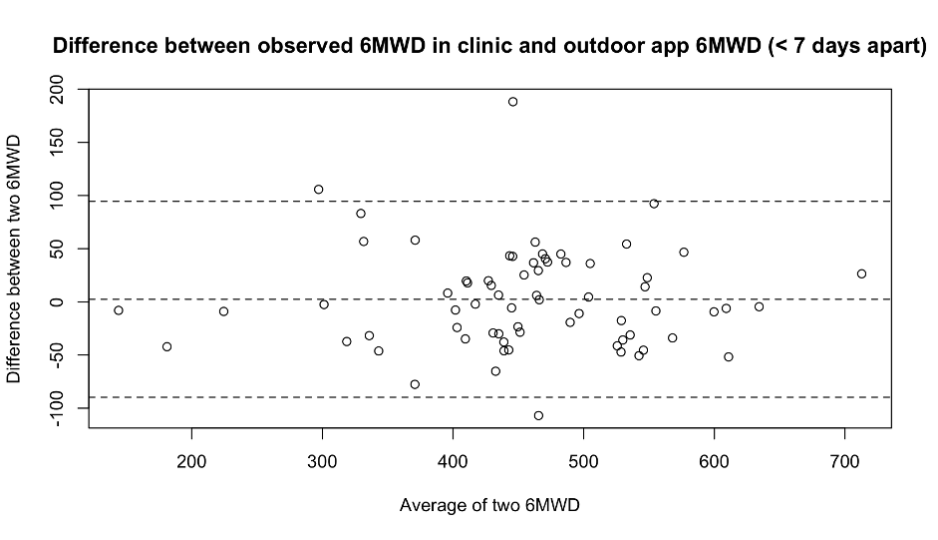
Bland-Altman plot of the difference between the 6-minute walk test distance as observed by the physiologist during a 6-minute walk test in clinic and as measured by the app in outdoor mode within 7 days of the clinic test. 6MWD: 6-minute walk test distance.

We compared pairs of 6MWD estimates where one was the result of an observation done by a physiologist in a clinic and the other was measured by the app during an outdoor test performed within 7 days of the clinic test. We analyzed 69 pairs belonging to 22 patients. Statistics for the differences and Bland-Altman plots are provided in Table 2 and Figure 4, respectively.

**Table 2:** Differences between 69 pairs containing a 6-minute walk test distance (6MWD) estimated by a physiologist during a clinic and a 6MWD measured by the app in outdoor mode within 7 days of the clinic test.

Characteristic	Value
Difference (m), mean (SD)	2.45 (47.0)
Difference (% of total length), mean (SD)	–0.18 (11.04)
Difference (m), median (min-max)	–4.55 (–106.91 to 188.24)
Difference (% of total length), median (min-max)	–0.83 (–26.35 to 34.86)
Correlation, (*P* value)	0.89 (<.001)
Percentages of difference below 42 m (%)	65
Lower and upper limits of agreement (m)	–89.67 to 94.57

We visually inspected the GPS traces of the 3 cases where the absolute difference was above 100 m and observed that, in 2 of those cases, the patient walked in small circles or performed numerous U-turns, which affects the accuracy of the distance estimation algorithm [14].

### Test-Retest Reliability of Community-Based 6MWT

**Figure 5:**
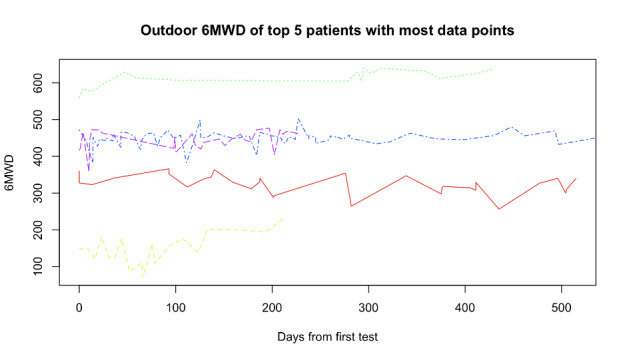
Outdoor, community-based 6-minute walk test distances over time of the top 5 patients who contributed with the most 6-minute walk tests. 6MWD: 6-minute walk test distance.

The 6MWD estimates provided at the end of community-based outdoor 6MWTs were stable over time (examples shown in Figure 5). To quantify this, we analyzed 89 pairs of outdoor tests performed by the same patient no more than 7 days apart. These pairs belonged to 10 patients in total. Statistics of the differences are reported in Table 3 together with intraclass correlation coefficient and standard error of measurement.

**Table 3:** Differences between the 6-minute walk test distances (6MWDs) of 89 pairs of community-based 6MWDs measured by the app in outdoor mode no more than 7 days apart.

Characteristic	Value
Difference (m), mean (SD)	1.80 (36.97)
Difference (% of total length), mean (SD)	0.72 (10.13)
Difference (m), median (min-max)	0.95 (–91.69 to 107.90)
Difference (% of total length), median (min-max)	0.20 (–25.64 to 35.26)
Correlation, (*P* value)	0.93 (<.001)
Percentages of difference below 42 m (%)	80
Intraclass correlation	0.91
Standard error of measurement (m)	36.97
Coefficient of variation (%), mean	12.45

We manually inspected the only pair of tests that presented a difference of 6MWD higher than 100 m and it appeared that in one of the tests, the user walked in a narrow path with U-turns.

### Compliance, Usability, and Acceptance

**Figure 6:**
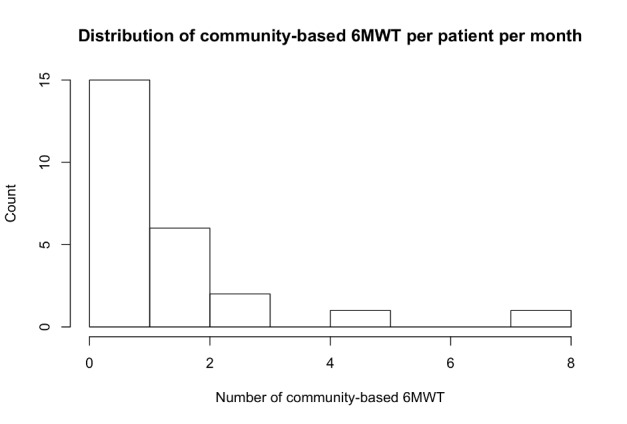
Histogram of the number of community-based 6-minute walk tests per patient per month. 6MWT: 6-minute walk test.

All patients who completed the study sent the results of at least one community-based 6MWT during the study, and 48% (12/25) of those who completed the study provided at least 1 6MWT result per month. The distribution of the number of community-based 6MWTs performed per patient per month is shown in Figure 6.

A total of 18 patients completed the usability and acceptance questionnaire. Likert scales to rate usability from 1 (very negative opinion) to 5 (very positive opinion) were all marked 3 and above. Also, the perceived impact of the app scored highly, with all average scores above 3. The answers provided to the acceptance questionnaire were above 3 on average except the question related to the perceived vulnerability construct (“I do not think my doctor understands how I feel doing my daily activities”). The exact wording of the questions and statistics of the answers are provided in Multimedia Appendix 2.

The questionnaire concluded with open questions about negative and positive aspects of the app and possible improvements. Answers with similar topics were grouped by one researcher and summarized.

Patients were happy with the app because it is easy to use (9/18), allows patients to perform the test within their community instead of having to go to the hospital (4/18), indicates the fitness level and oxygen saturation (4/18), and invites patients to walk regularly (1/18).

Reasons for concern were that it sometimes takes too long or fails to send the data (4/18), some elements of the user interface (eg, how to cancel a test) are not clear (2/18), patients forget to use it (1/18), log-in procedure is complicated (1/18), if a test fails it must be repeated (1/18), user interface could be more colorful and less medical (1/18), it does not accurately compute steps (1/18), pulse oximeter does not work correctly in cold weather (1/18), and some patients find it hard to walk for 6 minutes (1/18).

As for improvements, patients suggested providing charts about pulse oximetry on the app (3/18), integrate a wearable device (1/18), improve the feedback on incorrect log-in (1/18), make the detection of GPS satellites faster (1/18), improve user interface (1/18), and give the possibility of shortening the time of the test (1/18).

We interviewed 12 patients with a semistructured format (questions in Multimedia Appendix 3) on their last day in the study. Both notes taken during the interviews and transcripts were later analyzed by one researcher who grouped similar topics and summarized the feedback.

Motivating factors for using the app were self-monitor health status (7/12), pushing oneself to walk more (2/12), wanting to help research (2/12) and the feeling of being monitored by caregivers (1/12). In order to increase use, 1 patient suggested including reminders. The app was seen as useful (7/12), easy to use (3/12) and allowing better 6MWT than in the clinic because of not having to turn around (1/12).

Factors that contributed to not using the app were lack of time (3/12), weather (either too cold or too warm; 3/12), laziness (1/12), concerns about privacy (ie, not wanting to be seen walking holding the phone and pulse oximeter; 1/12), or lack of interest because of improved health conditions (1/12). Technical issues were reported about the app being too slow sending data (5/12), short battery life of the pulse oximeter (2/12), or inaccuracy compared to distance reported on Google Maps (1/12). Patients explicitly reported the app or technology not being an issue for motivation (1/12) nor concerns about safety (8/12) or data protection (7/12).

Most patients felt the app made them feel more connected with the caregiver who could review the data (10/12), and some would even trust it to substitute their periodic appointment with the doctor (5/12). In terms of suggestions, patients thought they wanted to have more comprehensive data about past tests (eg, pulse oximetry; 3/12), integration with fitness trackers and apps (3/12), reminders (2/12), and a simpler Borg scale (1/12). More than half of the patients (7/12) indicated they would be willing to use the app after the end of the study.

### Reliability of Pulse Oximetry

**Figure 7:**
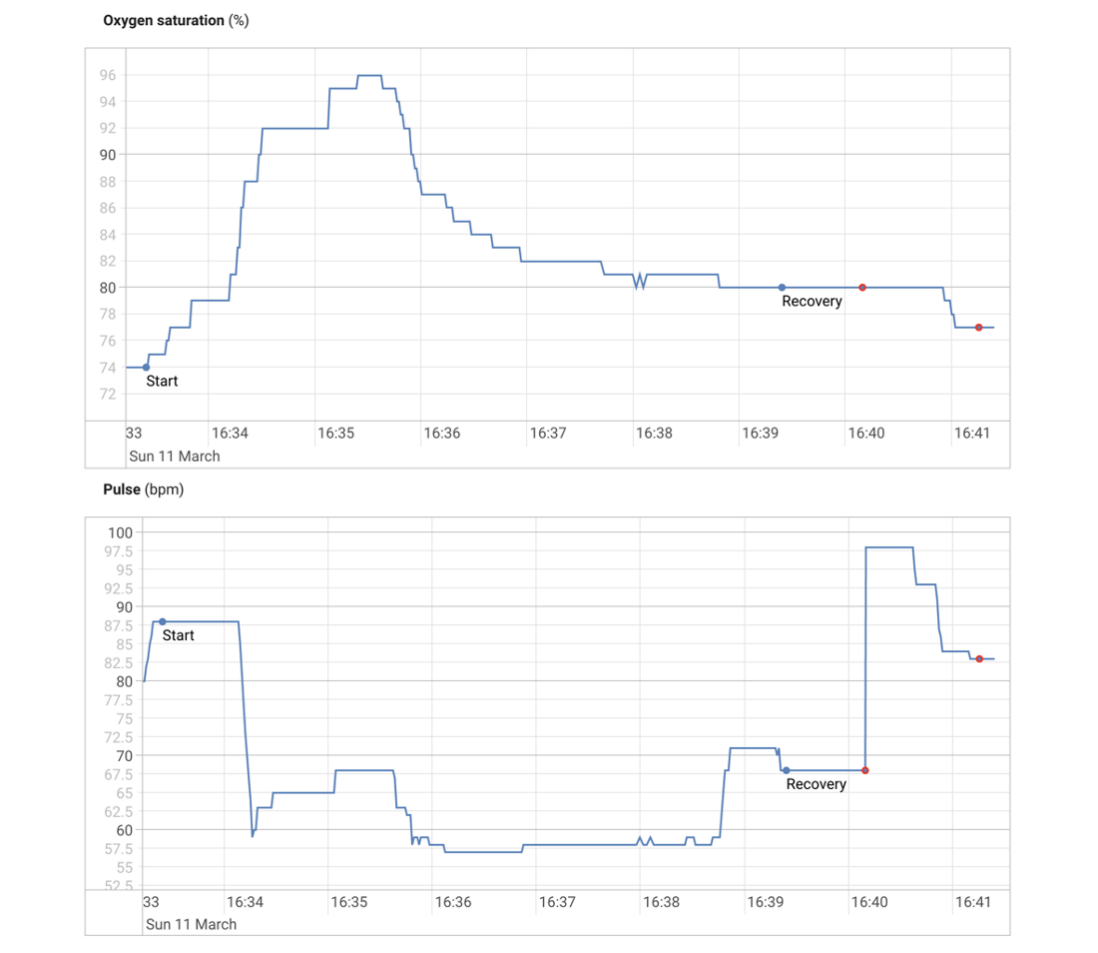
SpO_2_ and heart rate for a test where the SpO_2_ increases during exertion and the heart rate decreases. SpO_2_: peripheral oxygen saturation.

While monitoring data quality in the study, we observed that in 33.6% (130/387) of tests where pulse oximetry was recorded, the pulse oximeter presented a peripheral oxygen saturation (SpO_2_) value higher 3 minutes after the start of the test than at baseline. Likewise, 28.2% (109/387) of tests showed a decrease in the heart rate (HR) after 3 minutes with respect to the baseline. In order to explain this unexpected behavior, we analyzed the traces of SpO_2_ and HR for some of these tests (an example of such a test is shown in Figure 7). We hypothesized that this behavior was due to how the pulse oximeters were used; therefore, during clinic 6MWTs, we began collecting pulse oximetry from both the patient app, which uses the PC-68B, and the physiologist app, which can connect to the WristOx_2_ 3150. In addition, we asked one patient to run the app on two independent phones when doing community-based 6MWTs, each phone using one of the two types of pulse oximeters.

**Figure 8:**
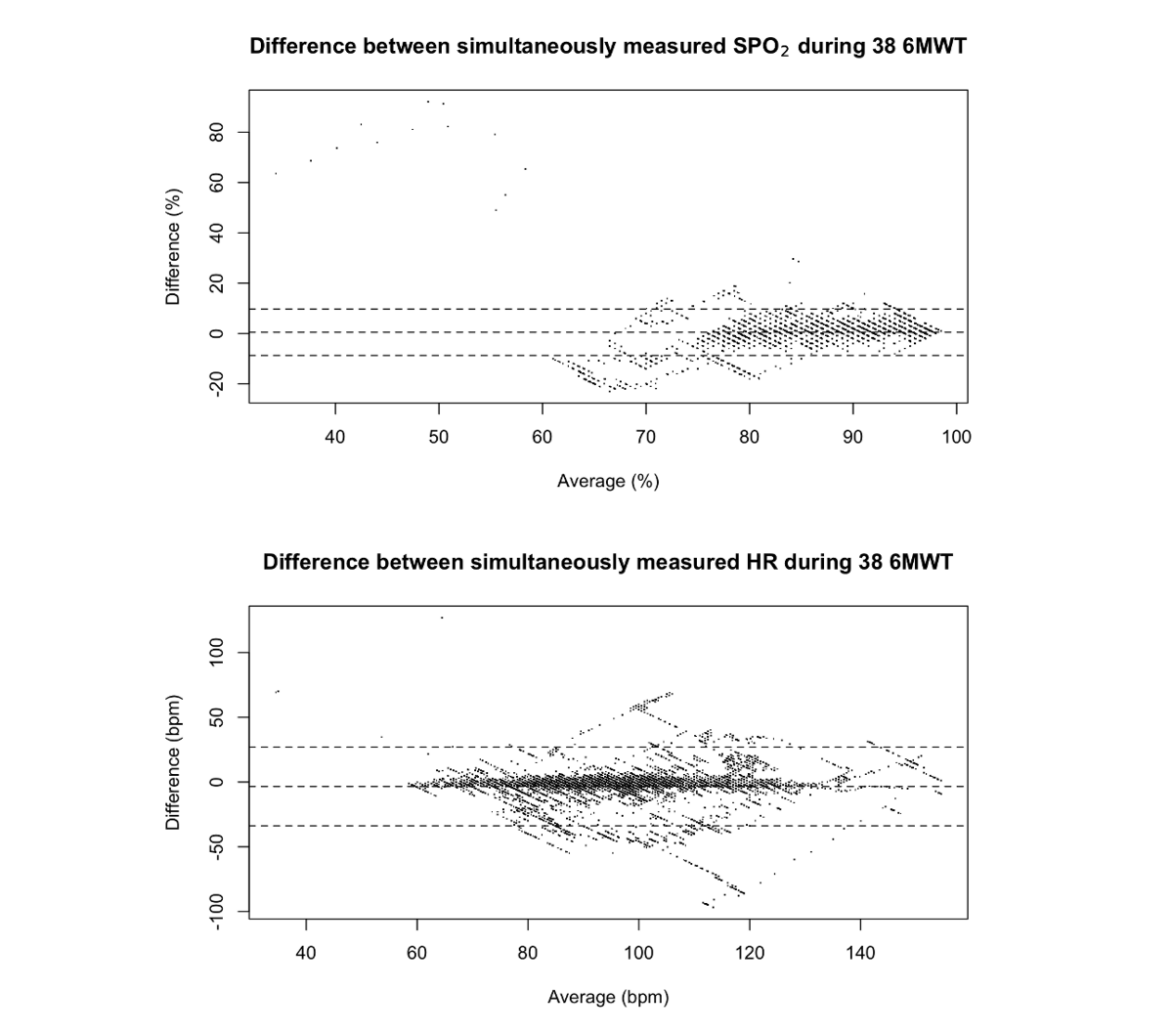
Bland-Altman plots of the differences between 19,356 matched samples of SpO_2_ and heart rate values measured simultaneously by two different pulse oximeters during 38 6-minute walk tests (6MWTs). SpO_2_: peripheral oxygen saturation.

With this strategy, we retrieved pairs of pulse oximetry samples (SpO_2_ and HR) from 38 tests. Samples with the shortest time differences (5 seconds or less) were matched and interpolated, thereby obtaining 19,279 matched samples to be compared. Figure 8 shows the Bland-Altman plot. The lower and upper limits of agreement are –8.72% and 9.73% for SpO_2_ and –33.87 and 26.97 bpm for HR.

## Discussion

### Accuracy of the App 6MWT in Indoor Mode

Compared to the lab tests of the same app reported in Salvi et al [14], the tests with patients’ phones showed higher variability in the differences between the distance measured by the physiologist and the distance measured by the phone (–2.01 [SD 7.84] m in lab tests vs 14.6 [SD 75.48] m in our study). The distribution of error by phone does not suggest any systematic bias in some phones; therefore, the inaccuracy must be due to specific test conditions that are not well tolerated by the distance estimation algorithm.

The Bland-Altman analysis shows that the limits of agreement (–133.35 and 162.55 m) are above the clinically significant threshold of 42 m; therefore, the app-based indoor measurement cannot be considered equivalent to the physiologist observation. Nonetheless, the analysis also shows that in 83% of the measurements the difference was below that threshold, which suggests that the algorithm fails sporadically but when it does, it introduces a high error, thus skewing the statistics.

### Validity of Community-Based Outdoor 6MWD

Our results show that the community-based, outdoor 6MWT correlated highly with the 6MWT performed in the clinic. This is similar to what is reported by Brooks et al [13] where a similar app is used (our 0.89 correlation coefficient vs their 0.88), although, in Brooks et al [13], a lower standard deviation of the difference is reported (26 m vs our 47 m).

While the high correlation confirms the general validity of the approach, Bland-Altman analysis shows that high differences can exist between clinic- and community-based measurements. These must be taken into account when interpreting the measured 6MWD, especially because the standard deviation of the difference was 47 m, which is above the clinically significant threshold. These differences can be explained by a combination of three factors: (1) inaccuracy of the technology: lab tests show that this amounts to up to 20 m of the standard deviation [14]; (2) variations in 6MWD when the test is performed on different days: analysis of conventional 6MWT in PAH shows a standard deviation of the difference of about 20 m [7,21]; and (3) some tests performed without following the instructions may have passed through our data quality assurance measures: the algorithm is known to lose accuracy when the test is performed in confined spaces and/or in the presence of many curves, and this was confirmed at least in 2 of the 3 cases where the difference was above 100 m.

### Test-Retest Reliability of Community-Based 6MWT

Consecutive tests performed within 7 days showed a high correlation (0.93). Compared to a similar app [13], we obtained a higher coefficient of variation (our 12.45% vs their 4.6%). Compared to the test-retest reliability of the GPS-method reported by Wevers et al [10], we obtained a similarly high intraclass correlation (our 0.91 vs their 0.96) but a higher standard error of measurement (our 36.97 m vs their 18.1 m). The standard deviation of the measurement error of the app as calculated in lab settings, 18.56 m [14], indicates that part of the standard error of measurement within consecutive measurements can be justified by the inaccuracy of the instrument. Nonetheless, it has to be noted that this value is lower than the clinically significant threshold of 42 m, as about 80% of the differences were below that threshold.

### Compliance, Usability, and Acceptance

While 100% of patients were able to use the app at least once during the 6-month duration of the study, only 48% followed the recommendation to perform 1 or more community-based 6MWTs per month. Low compliance is a known issue in telehealth studies [22]. As interviews revealed, low compliance in our cohort was due to lack of time or motivation or patients forgetting to use the app. It should be observed, however, that compliance varied significantly from patients who performed only one community-based test in the whole study to patients who performed more than 7 tests per month.

A possible explanation for low motivation in some patients could be the low perceived vulnerability (ie, patients feeling that they do not need further monitoring/follow-up in addition to regular clinics). This is particularly relevant within the context of this study as patients were informed that the study investigator (a cardiologist) would not use the collected data for any clinical decision making. Other external factors like weather conditions or not wanting to be seen using the app and the pulse oximeter may have also contributed.

The results of the questionnaire and the interviews show that the app was easy to use and well accepted. This is in agreement with other similar apps, like the one reported in Brooks et al [13]. In addition, the app was perceived as safe, secure (from the data protection perspective), and useful. Patients particularly appreciated the knowledge of their health being monitored either by themselves or the clinicians. A few usability and technical issues related to lack of control for some parts of the test (eg, difficulty in restarting the test) or data transmission (too slow and lacking feedback about connection status) were identified. However, these technical issues were not reported as a barrier for the use of the app.

In terms of improvements, patients suggested including reminders of scheduled tests, which could also boost compliance, integrating the app with other fitness apps and wearables, and providing more detailed data, especially about past tests.

### Reliability of Pulse Oximetry

The inaccuracy of pulse oximetry during walking is known in the literature [23-25] and may be due to arterial flow being significantly affected by motion. The two pulse oximeters employed in our studies were each certified as medical devices, and they each showed decreasing HR and increasing SpO_2_ at times during walking. Comparison of the data produced by the devices suggests that their values differed significantly (–8.72% and 9.73% for SpO_2_ and –33.87 and 26.97 bpm for HR), which is above what would be justified by the confidence interval reported by the manufacturers (±3 for both SpO_2_ and HR). We could not determine which of the devices was more accurate.

### Principal Findings

The main findings of this study are that, while the app-based indoor 6MWTs cannot be considered equivalent to the conventional one, the outdoor, community-based 6MWT measurements are strongly correlated with those performed in clinic and are repeatable. These community-based tests can be considered as a valid complement to conventional 6MWTs especially because they can be performed more frequently and at the patient’s convenience, but patients must be instructed to use the app correctly to avoid inaccuracies.

Results also show that even if the app has proven to be usable and well accepted, its usefulness should be made explicit to patients to increase their engagement. Additionally, the use of conventional pulse oximetry should not be relied on in a 6MWT, at least during the walking stage.

### Implications for Future Research

Future versions of this system would benefit from technical improvements. The insufficient accuracy shown in the indoor scenario suggests that new algorithmic approaches should be explored to make the measurement more robust. Algorithms used for dead-reckoning of pedestrians may offer a valid strategy [26]. In addition, the algorithm used for the outdoor scenario is penalized when the user walks over narrow or curved paths. Data fusion techniques combining GPS and dead-reckoning could possibly improve accuracy in those nonideal conditions. Further data analysis could be performed to understand if oxygen saturation measurements from different pulse oximeters converge when patients are not moving. It is likely that the measurements are more reliable at least before the start and after the end of the test. If pulse oximetry during the test is desirable, motion-resistant pulse oximeters may provide a reliable solution for this scenario [27]. Compliance to the testing regime could be boosted by simple techniques like reminders. The 6MWD could be linked or complemented with data collected by wearables and fitness trackers, which may provide a less obtrusive way of measuring exercise capacity.

In terms of clinical significance, longitudinal studies in which the data collected by the app are used in clinical practice are needed to understand the usefulness of these tests. For example, could remote monitoring improve follow-up and support titration of medications? We also expect that clinical use of the app would affect compliance. How would patients behave when they know their use of the app could lead to different health outcomes?

### Limitations

This pilot study involved 30 patients and was not aimed at obtaining statistical significance; larger cohorts would be needed to confirm the results. It is also important to observe that our manual data quality assurance strategy was not always consistent and that an unestimated number of outdoor tests performed in the wrong conditions were included in the statistics.

### Conclusions

Our app-based outdoor 6MWT in community settings is valid, repeatable, and well accepted by patients. Its use could complement or potentially substitute conventional 6MWTs in clinics. The same app, however, is not accurate enough for clinical use when used indoors.

## Appendix

Multimedia Appendix 1 Examples of valid and invalid tests.

Multimedia Appendix 2 Usability and acceptance questionnaire.

Multimedia Appendix 3 Interview questions.

## Abbreviations

6MWD: 6-minute walk test distance
6MWT: 6-minute walk test
HR: heart rate
PAH: pulmonary arterial hypertension
SpO_2_: peripheral oxygen saturation
