# Supplementary material for: App-Based Versus Standard Six-Minute Walk Test in Pulmonary Hypertension: Mixed Methods Study
Source: JMIR Mhealth Uhealth. 2021 Jun 7;9(6):e22748. doi: 10.2196/22748 (PMC8218218; doi:10.2196/22748)
Supplement: Multimedia Appendix 1 [file mhealth_v9i6e22748_app1.docx]

## Multimedia Appendix 1. Examples of valid and invalid tests

The SWMT app sent all the location points collected during the outdoor 6MWT. It was possible to visualise the sequence of location points on the web interface. Traces were reviewed by the research team on a weekly basis. Given that the algorithm that computes distance from coordinates is more accurate when the walked path is straight or has gentle curves, paths that presented more than 3 narrow or U-turns were marked as “not valid”. Examples of good quality and bad quality traces are shown in figure a1.1.


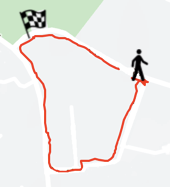

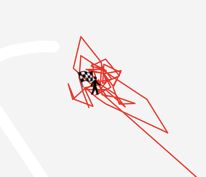


(a) (b)

Figure a1.1: examples of location traces collected during outdoor 6MWT. (a) example of good-quality trace, the path is clear and gently curved. (b) example of a bad-quality test: the path is not clear; the user probably walked in narrow circles around one location.

For indoor tests, the quality was assessed by looking at the traces of the phone’s embedded compass. In most phones, the orientation signal is derived using a combination of the accelerometer and the magnetometer. This signal may be affected by lack of proper calibration and noise. This noise and low amplitude of this signal, in turns, may affect the algorithm used to detect U-turns. While the app requested users to perform a calibration procedure before each indoor test, the data showed that sometimes this was not enough to guarantee the correct functioning of the algorithm. Examples are provided in figure 11.

1.
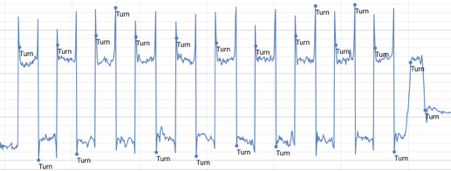

2.
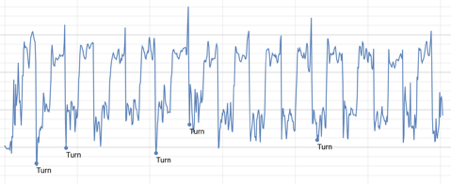


Figure 11. Examples of good and bad-quality compass signals. The compass provides the number of degrees from the North, from 0 to 360 degrees. (a) shows a good-quality signal: the amplitude of the noise is negligible compared to the variation of the direction during the test and all turns are identified. (b) shows a bad-quality signal, where substantial noise is visible and only a few U-turns were identified by the algorithm.
