# Supplementary material for: App-Based Versus Standard Six-Minute Walk Test in Pulmonary Hypertension: Mixed Methods Study
Source: JMIR Mhealth Uhealth. 2021 Jun 7;9(6):e22748. doi: 10.2196/22748 (PMC8218218; doi:10.2196/22748)
Supplement: Multimedia Appendix 2 [file mhealth_v9i6e22748_app2.docx]

## Multimedia Appendix 2. Usability and acceptance questionnaire

The following are the questions presented in the usability and acceptance questionnaire. The questions were adapted from [18] and [19]. Possible choices for answers and statistics for the received answers are provided for multiple-choice type questions.

Question: Is the app interesting to use? Does it present its information in an interesting way compared to other similar apps?

Possible choices: 1 Not interesting at all, 2 Mostly uninteresting, 3 OK, neither interesting nor uninteresting; would engage user for a brief time (< 5 minutes), 4 Moderately interesting; would engage user for some time (5-10 minutes total), 5 Very interesting, would engage user in repeat use

Average score (± standard deviation): 4.17 (±0.99)

Question: Is the app content (visuals, language, design) appropriate for the target audience?

Possible choices: 1 Completely inappropriate, unclear or confusing, 2 Mostly inappropriate, unclear or confusing, 3 Acceptable but not specifically designed for the target audience. May be inappropriate/ unclear/confusing at times, 4 Designed for the target audience, with minor issues, 5 Designed specifically for the target audience, no issues found

Average score: 4.33 (±0.59)

Question: How accurately/fast do the app features (functions) and components (buttons/menus) work?

Possible choices: 1 App is broken; no/insufficient/inaccurate response (e.g. crashes/bugs/broken features, etc.), 2 Some functions work, but lagging or contains major technical problems, 3 App works overall. Some technical problems need fixing, or is slow at times, 4 Mostly functional with minor/negligible problems, 5 Perfect/timely response; no technical bugs found, or contains a ‘loading time left’ indicator (if relevant)

Average score: 4.22 (±0.65)

Question: How easy is it to learn how to use the app; how clear are the menu labels, icons and instructions?

Possible choices: 1 No/limited instructions; menu labels, icons are confusing; complicated, 2 Takes a lot of time or effort, 3 Takes some time or effort, 4 Easy to learn (or has clear instructions), 5 Able to use app immediately; intuitive; simple (no instructions needed)

Average score: 4.17 (±0.71)

Question: Does moving between screens make sense? Does the app have all necessary links between screens?

Possible choices: 1 No logical connection between screens at all /navigation is difficult, 2 Understandable after a lot of time/effort, 3 Understandable after some time/effort, 4 Easy to understand/navigate, 5 Perfectly logical, easy, clear and intuitive screen flow throughout, and/or has shortcuts

Average score: 4.28 (±0.57)

Question: Is arrangement and size of buttons, icons, menus and content on the screen appropriate?

Possible choices: 1 Very bad design, cluttered, some options impossible to select, locate, see or read, 2 Bad design, random, unclear, some options difficult to select/locate/see/read, 3 Satisfactory, few problems with selecting/locating/seeing/reading items, 4 Mostly clear, able to select/locate/see/read items, 5 Professional, simple, clear, orderly, logically organised

Average score: 4.56 (±0.51)

Question: How high is the quality of graphics used for buttons, icons, menus and content?

Possible choices: 1 Graphics appear amateur, very poor visual design - disproportionate, stylistically inconsistent, 2 Low quality/low resolution graphics; low quality visual design – disproportionate, 3 Moderate quality graphics and visual design (generally consistent in style), 4 High quality/resolution graphics and visual design – mostly proportionate, consistent in style, 5 Very high quality/resolution graphics and visual design - proportionate, consistent in style throughout

Average score: 4.11 (±0.58)

Question: How good does the app look?

Possible choices: 1 Ugly, unpleasant to look at, poorly designed, clashing, mismatched colours, 2 Bad – poorly designed, bad use of colour, visually boring, 3 OK – average, neither pleasant, nor unpleasant, 4 Pleasant – seamless graphics – consistent and professionally designed, 5 Beautiful – very attractive, memorable, stands out; use of colour enhances app features/menus

Average score: 3.83 (±0.51)

Question: Is app content correct, well written, and relevant to the goal/topic of the app?

Possible choices: N/A There is no information within the app, 1 Irrelevant/inappropriate/incoherent/incorrect, 2 Poor. Barely relevant/appropriate/coherent/may be incorrect, 3 Moderately relevant/appropriate/coherent/and appears correct, 4 Relevant/appropriate/coherent/correct, 5 Highly relevant, appropriate, coherent, and correct

Average score: 4.22 (±0.43)

Question: Is the information within the app comprehensive but concise?

Possible choices: N/A There is no information within the app, 1 Minimal or overwhelming, 2 Insufficient or possibly overwhelming, 3 OK but not comprehensive or concise, 4 Offers a broad range of information, has some gaps or unnecessary detail; or has no links to more information and resources, 5 Comprehensive and concise; contains links to more information and resources

Average score: 4.24 (±0.66)

Question: Is visual explanation of concepts – through charts/graphs/images/videos, etc. – clear, logical, correct?

Possible choices: N/A There is no visual information within the app (e.g. it only contains audio, or text), 1 Completely unclear/confusing/wrong or necessary but missing, 2 Mostly unclear/confusing/wrong, 3 OK but often unclear/confusing/wrong, 4 Mostly clear/logical/correct with negligible issues, 5 Perfectly clear/logical/correct

Average score: 4.22 (±0.55)

Question: Would you recommend this app to people who might benefit from it?

Possible choices: 1 Not at all I would not recommend this app to anyone, 2 There are very few people I would recommend this app to, 3 Maybe There are several people I would recommend this app to, 4 There are many people I would recommend this app to, 5 Definitely I would recommend this app to everyone

Average score: 4.33 (±0.97)

Question: How many times do you think you would use this app in the next 12 months if it was relevant to you?

Possible choices: 1 None, 2 1-2, 3 3-10, 4 10-50, 5 >50

Average score: 3.83 (±0.92)

Question: Would you pay for this app?

Possible choices: 1 Definitely not, 2, 3, 4, 5 Definitely yes

Average score: 3.00 (±1.17)

Question: What is your overall rating of the app?

Possible choices: 1 ★ One of the worst apps I’ve used, 2 ★ ★, 3 ★ ★ ★ Average, 4 ★ ★ ★ ★, 5 ★ ★ ★ ★ ★ One of the best apps I've used

Average score: 3.94 (±0.66)

Question: This app has increased my awareness of the importance of letting the doctor know how I feel when exercising.

Possible choices:, 1 Strongly disagree, 2 Disagree, 3 Neither agree nor disagree, 4 Agree, 5 Strongly Agree

Average score: 3.67 (±0.84)

Question: This app has increased my knowledge/understanding of keeping my doctor informed of my exercise capacity.

Possible choices: 1 Strongly disagree, 2 Disagree, 3 Neither agree nor disagree, 4 Agree, 5 Strongly Agree

Average score: 3.83 (±0.86)

Question: The app has changed my attitudes towards increasing the amount of exercise I do.

Possible choices: 1 Strongly disagree, 2 Disagree, 3 Neither agree nor disagree, 4 Agree, 5 Strongly Agree.

Average score: 3.28 (±0.75)

Question: The app has increased my intentions/motivation to exercise.

Possible choices: 1 Strongly disagree, 2 Disagree, 3 Neither agree nor disagree, 4 Agree, 5 Strongly Agree.

Average score: 3.33 (±0.69)

Question: Use of this app will increase the amount of exercise I do.

Possible choices: 1 Strongly disagree, 2 Disagree, 3 Neither agree nor disagree, 4 Agree, 5 Strongly Agree.

Average score: 3.28 (±0.57)

Question: I plan to use this app in the next 3 months.

Possible choices: 1 Strongly disagree, 2 Disagree, 3 Neither agree nor disagree, 4 Agree, 5 Strongly Agree.

Average score: 3.94 (±0.73)

Question: I like the idea of using this app.

Possible choices: 1 Strongly disagree, 2 Disagree, 3 Neither agree nor disagree, 4 Agree, 5 Strongly Agree.

Average score: 3.83 (±0.79)

Question: I have the knowledge necessary to use this app.

Possible choices: 1 Strongly disagree, 2 Disagree, 3 Neither agree nor disagree, 4 Agree, 5 Strongly Agree.

Average score: 4.29 (±0.69)

Question: Using services like this app will make my life more convenient

Possible choices: 1 Strongly disagree, 2 Disagree, 3 Neither agree nor disagree, 4 Agree, 5 Strongly Agree.

Average score: 3.81 (±0.66)

Question: Overall, this app is easy to use.

Possible choices:, 1 Strongly disagree, 2 Disagree, 3 Neither agree nor disagree, 4 Agree, 5 Strongly Agree.

Average score: 4.17 (±0.62)

Question: I do not think my doctor understands how I feel doing my daily activities.

Possible choices: 1 Strongly disagree, 2 Disagree, 3 Neither agree nor disagree, 4 Agree, 5 Strongly AgreePossible choices

Average score: 2.56 (±1.26)

Question: If they did understand, this would be a significant improvement.

Possible choices: 1 Strongly disagree, 2 Disagree, 3 Neither agree nor disagree, 4 Agree, 5 Strongly Agree.

Average score: 3.80 (±0.77)

Question: I think the app would help them understand

Possible choices: 1 Strongly disagree, 2 Disagree, 3 Neither agree nor disagree, 4 Agree, 5 Strongly Agree.

Average score: 3.83 (±0.51)

Question: I am able to use the app without much effort.

Possible choices: 1 Strongly disagree, 2 Disagree, 3 Neither agree nor disagree, 4 Agree, 5 Strongly Agree.

Average score: 4.00 (±0.77)

Question: I have to spend effort on learning how to use the SMWT app.

Possible choices: 1 Strongly disagree, 2 Disagree, 3 Neither agree nor disagree, 4 Agree, 5 Strongly Agree.

Average score: 2.11 (±0.90)

Question: Things I liked about this app:

Answer type: free text

Question: Things I disliked about this app:

Answer type: free text

Question: Suggested improvements and further comments:

Answer type: free text
