# Supplementary material for: App-Based Versus Standard Six-Minute Walk Test in Pulmonary Hypertension: Mixed Methods Study
Source: JMIR Mhealth Uhealth. 2021 Jun 7;9(6):e22748. doi: 10.2196/22748 (PMC8218218; doi:10.2196/22748)
Supplement: Multimedia Appendix 3 [file mhealth_v9i6e22748_app3.docx]

## Multimedia Appendix 3. Interview questions

The questions of the interviews were partially based on the acceptance model proposed in [20], of which, we particularly adopted the constructs of “perceived usefulness”, the “perceived privacy risk” and the “perceived performance risk”.

- (If the patient was not compliant) I see that you did not use the app very frequently. Was there anything that prevented you from using it?
- (If the patient was overall compliant) I see that you used the app quite regularly. What motivated you?
- Is there anything that would motivate you to use the app further?
- Did you find the app useful?
- Do you have any concerns about your health when using the app?
- Do you have any concerns about your data not being protected as it should?
- Are there any technical issues with the app that could be improved?
- Is there anything we could add or remove from the app to make it more useful?
- Do you think using the app would improve your outpatient appointment experience?
- Would you trust this technology to substitute your periodic appointment with your doctor?
- Would you want to continue to use the app?
- Do you have anything else you would like to ask, or any other feedback or questions about the app?
